# Acute Exposure to Cigarette Smoking Followed by Myocardial Infarction Aggravates Renal Damage in an *In Vivo* Mouse Model

**DOI:** 10.1155/2017/5135241

**Published:** 2017-10-22

**Authors:** Firas Kobeissy, Abdullah Shaito, Abdullah Kaplan, Lama Baki, Hassan Hayek, Carole Dagher-Hamalian, Ali Nehme, Rana Ghali, Emna Abidi, Ahmad Husari, Asad Zeidan, Fouad A. Zouein, Kazem Zibara

**Affiliations:** ^1^Department of Biochemistry and Molecular Genetics, Faculty of Medicine, American University of Beirut, Beirut, Lebanon; ^2^Department of Biological and Chemical Sciences, Lebanese International University, Beirut, Lebanon; ^3^Department of Pharmacology and Toxicology, American University of Beirut, Beirut, Lebanon; ^4^ER045, PRASE, DSST, Lebanese University, Beirut, Lebanon; ^5^CNRS UPR9002, Institute of Molecular and Cellular Biology, Strasbourg University, Strasbourg, France; ^6^Department of Pathology, School of Medicine, Lebanese American University, Beirut, Lebanon; ^7^GICC, UMR 7292, CNRS, Université François Rabelais de Tours, Tours, France; ^8^Department of Anatomy, Cell Biology and Physiological Sciences, American University of Beirut, Beirut, Lebanon; ^9^Department of Basic Medical Sciences, College of Medicine, Qatar University, Doha, Qatar; ^10^Biology Department, Faculty of Sciences-I, Lebanese University, Beirut, Lebanon

## Abstract

Cigarette smoking (S) is a risk factor for progressive chronic kidney disease, renal dysfunction, and renal failure. In this study, the effect of smoking on kidney function was investigated in a mouse model of myocardial infarction (MI) using 4 groups: control (C), smoking (S), MI, and S+MI. Histological analysis of S+MI group showed alterations in kidney structure including swelling of the proximal convoluted tubules (PCTs), thinning of the epithelial lining, focal loss of the brush border of PCTs, and patchy glomerular retraction. Molecular analysis revealed that nephrin expression was significantly reduced in the S+MI group, whereas sodium-hydrogen exchanger-1 (NHE-1) was significantly increased, suggesting altered glomerular filtration and kidney functions. Moreover, S+MI group, but not S alone, showed a significant increase in the expression of connective tissue growth factor (CTGF) and fibrotic proteins fibronectin (FN) and *α*-smooth muscle actin (SMA), in comparison to controls, in addition to a significant increase in mRNA levels of IL-6 and TNF-*α* inflammatory markers. Finally, reactive oxygen species (ROS) production was significantly accentuated in S+MI group concomitant with a significant increase in NOX-4 protein levels. In conclusion, smoking aggravates murine acute renal damage caused by MI at the structural and molecular levels by exacerbating renal dysfunction.

## 1. Introduction

Cardiovascular diseases (CVD) are the leading cause of mortality worldwide, in both men and women [1, 2]. In the USA, 1 person dies every 37 seconds due to CVD [3]. In low- and middle-income countries, increased CVD prevalence poses a dramatic burden [4]. Previous studies have demonstrated that CVD start at early childhood and is influenced throughout life by environmental, genetic, and modifiable risk factors [5]. Cigarette smoking (S), high blood pressure, high blood cholesterol, diabetes mellitus, sedentary lifestyle, and obesity are among the factors that influence CVD. It has been estimated that one fifth of all heart-related deaths in the USA are due to cigarette smoking [1–5].

According to the World Health Organization (WHO), the number of smokers has increased to almost a billion in 2012 [6] and smoking is estimated to account for 7 million deaths by 2020 [7]. Cigarette smoking (S) is the predominant form of tobacco smoking and is an addictive habit due to nicotine dependence [7]. Unfortunately, tobacco releases not only nicotine but also thousands of chemicals that are either toxic or carcinogenic [8]. S is a major health threat contributing to many diseases with high morbidity and mortality [2] such as lung cancer, bronchitis, emphysema, and CVD [9].

S is an important risk factor in the development of atherosclerosis, starting from endothelial cell (EC) dysfunction to acute clinical events. Early studies have shown that death from coronary heart disease (CHD) is more common in smokers than nonsmokers and that CVD is the major cause of smoking-related premature death [10]. In fact, while smoking alone doubles the risk of heart failure [11, 12], smokers are ~1.5 times more at risk of stroke than nonsmokers [13]. Finally, tobacco consumption increases the risk of peripheral artery disease more than heart diseases [10]. The correlation between CVD incidence and S could be attributed to the increase in oxidative stress due to the generation of free radicals during tobacco combustion [14]. Oxidative stress leads to the initiation of atherosclerosis in the vessels by impairing nitric oxide (NO) signaling, promoting macrophage adhesion to EC layer, among other mechanisms [15–17].

Free radicals are generated continuously; however, protective mechanisms in the body regulate their concentration leading to a well-balanced equilibrium [18]. Once this balance is compromised, an oxidative stress state occurs which associates with various pathophysiological conditions including autoimmune diseases, hypertension, cancer, diabetes, atherosclerosis, coronary heart disease, and heart failure [19, 20]. This is not surprising as ROS production is intimately associated with aberrant inflammatory signalling, elevated platelet activation, and increased leukocyte recruitment to the site of inflammation [21–23]. Importantly, it is now well documented that development of vascular diseases such as atherosclerosis or hypertension is associated with increased levels of ROS and prooxidants [24, 25].

Several studies showed that S induces renal tubular injury and glomerular damage, leading to kidney diseases and dysfunction [26]. In addition, nicotine was shown to reduce cell viability and to induce apoptosis in human renal proximal tubular epithelial cells [27]. Recently, we have shown that S-induced cardiac hypertrophy, vascular inflammation, and injury were attenuated by antioxidant supplementation in an animal model [28]. On the other hand, myocardial infarction (MI) enhances progressive renal damage through the cardiorenal interaction axis since reduced cardiac output may lead to reduced renal perfusion [29]. Consequently, this leads to compensatory renin-angiotensin-aldosterone system (RAAS) activation leading to hypertension and end-organ damage to the heart and kidneys [30–33]. The above clearly demonstrate that both S and MI can independently impair renal function. There are not much data on the interaction between S and MI in inducing renal damage. Moreover, it is not known whether S and MI lead to renal damage through similar or different pathways. Epidemiological studies showed that S is an independent risk factor in the development of chronic kidney diseases (CKDs) of different etiologies, including hypertension and diabetes [34–36]. However, the mechanisms by which S accelerates the progression of CKD are not well understood. In this study, we evaluated whether the combination of S and MI can aggravate the renal damage seen in the individual insult of S or MI. This was accomplished by looking at hallmarks of kidney damage such as increased deposition of kidney ECM, kidney fibrosis, and increased inflammation.

This study aims to mimic the clinical concept of smoking cessation post-MI following hospitalization by investigating the impact of acute smoke exposure on early kidney injury following acute cardiac injury. Indeed, we sought to determine the effect of S and MI on kidney function by measuring key functional proteins and proinflammatory and oxidative stress markers, in addition to alterations in kidney structure, in a mouse model of acute exposure to S. We hypothesize that S followed by MI aggravates the renal damage caused by S or MI alone, leading to renal dysfunction. Results of this study should raise awareness about S as a risk factor, especially when followed by MI.

## 2. Materials and Methods

### 2.1. Animals

This study was approved by the Institutional Animal Care and Utilization Committee (IACUCC number: 17-04-410) of the American University of Beirut (AUB) for the project entitled “Assessment of Renal Changes after Smoking and Myocardial Infarction: A Comparative Study.” Male C57BL6 mice weighing 20–25 g were purchased from Charles River Laboratories (Wilmington, MA, USA) and housed under pathogen-free conditions with constant temperature and humidity control at the AUB animal care facility. Mice had sterile bedding and ad libitum access to water and rodent feed. A total of 20 mice, at 16 weeks of age, were divided into 4 groups and used in this study. All surgical procedures were performed under deep anesthesia.

### 2.2. Experimental Design

A total of 20 mice, at 16 weeks of age, were used in this study. Four groups of 5 mice each were divided during the 2-week study period as follows: controls (C), smoking (S, 2 cigarettes per day for 7 days), myocardial infarction (MI, LAD ligation for 7 days), or both smoking and myocardial infarction (S+MI). For the last group, one week subsequent to S, MI was performed by permanently ligating the left anterior descending (LAD) coronary artery. Mice were then sacrificed one week post-MI.

### 2.3. Experimental Smoke Exposure *In Vivo* Model

Briefly, age-matched male C57BL/6J mice were exposed to cigarette smoke (S) using a cigarette smoking exposure apparatus (ONARES, CH Technologies, USA). This apparatus includes a smoke generator with a mixing/conditioning chamber and a “nose-only” rodent exposure carousel. It allows for exposure to mainstream smoke from a cigarette in conscious, restrained rodents. This system has been extensively used to study smoking-related diseases. Mice in the smoking groups (S, S+MI) were exposed to the smoke of 3R4F research-grade cigarettes (University of Kentucky, Lexington, KY, USA), which are scientifically prepared cigarettes concentrated with toxins and chemicals rendering the study timeline suitable to observe the effects of smoking on the mice. Cigarettes were placed into the cigarette puffer, and a peristaltic pump was used to generate puffs at a frequency of 1 puff/min, a duration of 2.5 seconds/puff, and a volume of 5 ml/puff. Animals received 10.9 mg total particle matter (TPM), 9.4 mg tar, and 0.726 mg nicotine per cigarette. The amount of TPM were measured on the smoke filters before and after the experiment. Mice received S twice daily for 1 week preceding MI. Previously, plasma cotinine measurements were conducted in the S group by competitive chemiluminescent immunoassay (Siemens Healthcare Diagnostics, Llanberis, UK) in order to verify systemic exposure to aerosol constituents using the nose-only exposure system. Animals were found not to be previously exposed to any substance other than clean air, and therefore the measured cotinine levels were strictly due to exposure during the protocol. Plasma cotinine measurements conducted for 5 animals in the S group after 1 day of exposure were found to be 76 ± 7.6 ng/ml [37].

### 2.4. Left Anterior Descending (LAD) Coronary Artery Ligation

Induction of MI by LAD ligation leads to the development of heart failure. Briefly, the mouse was placed on a heating pad to prevent hypothermia, and orotracheal intubation was performed by placing a needle into the trachea, connected to a mini ventilator (Harvard Apparatus). Following skin incision, pectoralis muscles were retracted and intercostal muscles carefully dissected. Small incisions were made in order to expose the left atrium (LA), left ventricle (LV), and the LAD coronary artery. MI was performed by ligation of the LAD coronary artery 1–3 mm from the tip of the LA, followed by closure of the thorax. Occlusion of LAD was confirmed by the appearance of a pale color in the anterior wall of the LV within a few seconds after ligation. Infarction was defined by blanching of the LV and ST elevation on the electrocardiogram using Indus MouseMonitor system. Before surgery, mice were given tramadol as analgesic then anesthetized with isoflurane (2-3% in oxygen). During recovery, mice were placed in a warm incubator and monitored closely until freely grooming. Mice were then monitored on a daily basis.

### 2.5. Echocardiography

After induction of MI, animals were checked every other day by ultrasounds, for 7 days, to assess cardiac remodeling. Briefly, transthoracic echocardiography was performed using SonixTouch ultrasound system (Ultrasonix Medical Corporation, Richmond, Canada) equipped with a high-frequency linear array transducer (L40-8). Mice were anesthetized with 1-2% isoflurane in an oxygen mix and placed on an electrical body temperature heated table. 2D (B-mode) images of the left ventricle were acquired from the parasternal long axis view in supine position. Indeed, parasternal long- and short-axis views were taken by directing the ultrasound beam at the midpapillary muscle level. Continuous interface of the anterior and posterior walls was visualized and well defined before measurements. The transducer was placed on the left thorax, and M-mode and 2D echocardiography images and mean calculations were obtained from three or more consecutive cardiac cycles. Body temperature, heart rate, and respiratory rate were continuously monitored throughout the procedure to adjust the depth of anesthesia. Linear internal measurements of the LV were acquired from the 2D parasternal long-axis images at the midpapillary level. Left ventricular volumes and ejection fraction were calculated according to a cube function formula [38].

### 2.6. Animal Tissue Harvesting

At the end of the experimental period, mice were anesthetized with isoflurane then euthanized by cervical dislocation. Kidneys were isolated and divided into 2 parts: either fixed in formalin and embedded in paraffin for histopathology examination or snap frozen in liquid nitrogen then stored at −80°C to be used later for RNA studies and protein analysis.

### 2.7. Protein Extraction and Quantification

Snap frozen kidneys were ground in liquid nitrogen by a mortar and pestle and then homogenized in lysis buffer (1X RIPA buffer; 250 mM Tris-HCl, 750 mM sodium chloride, 5% Igepal CA-630, 5% sodium deoxycholate, and 0.5% sodium dodecyl sulfate) containing protease and phosphatase inhibitors (1 mM PMSF, 1 mM benzamidine, 2 mM Na orthovanadate, 10 mM NaF, 1 mM Na pyrophosphate, 2 *μ*g/ml Leupeptin, 2 *μ*g/ml aprotinin, and 1 *μ*l/ml DTT), followed by a centrifugation at 12000 ×g for 10 min at 4°C. Total protein concentrations were then determined using “DC™ Protein Assay II” kit Bradford method (Bio-Rad, Hercules, CA, USA).

### 2.8. Western Blotting

Protein samples (30 *μ*g) were loaded into the wells of 10% SDS-PAGE gel and run until the dye front reached the bottom of the gel. Gels were then transferred to PVDF membranes at 4°C at 30–40 volts overnight. The membrane was then blocked in 5% fat-free milk, prepared in Tween PBS, for 1 h at RT. Proteins were detected by monoclonal primary antibodies against sodium hydrogen exchanger (NHE-1), nephrin, connective tissue growth factor (CTGF), fibronectin (FN), *α*-smooth muscle actin (SMA), NOX-4, and GAPDH (Sigma, USA); the latter was used to ensure equal loading of samples. Immunoblots were then probed with the appropriate secondary antibodies (anti-mouse 1 : 5000, anti-rabbit 1 : 10000) for 1 h at RT. Bands were detected with ECL chemiluminescence kit (Thermo Fisher scientific). Intensity of bands was then determined by densitometry, using the ImageJ software (https://imagej.nih.gov/ij/).

### 2.9. RNA Extraction

Snap frozen tissues were ground in liquid nitrogen by a mortar and pestle then total RNA was isolated using TRIzol according to manufacturer's instructions (Thermo Fisher Scientific, Grand Island, NY, USA). The quantity of RNA was measured using the NanoDrop® ND-1000 UV-Vis spectrophotometer. RNA purity was assessed using the absorbance ratio of 260 to 280 nm, where a value of 1.8–2.0 indicated good-quality RNA. Finally, RNAs were treated by Deoxyribonuclease I (Thermo, USA) to remove contaminating genomic DNA.

### 2.10. Real-Time qPCR

To quantify differences in mRNA expression, gene expression was monitored using SYBR^®^ Green PCR Master Mix (Bio-Rad, Hercules, CA, USA) to quantify the gene expression of interleukin-6 (IL-6) and tumor necrosis factor alpha (TNF-*α*) genes. GAPDH expression was used to normalize gene expression between the different samples. During RT step, cDNA was synthesized from 1 *μ*g RNA, using Revert Aid 1st Strand cDNA synthesis kit (Thermo, USA), followed by real-time PCR analysis in a CFX96 real-time PCR system (Bio-Rad, Germany). For the qPCR reaction, the synthesized cDNA was loaded in duplicates with 5 *μ*M of each of the forward and reverse primers of the gene of interest and mixed with SYBR Green. The PCR conditions were 95°C for 5 min, then 40 cycles of denaturation at 95°C for 10 sec, annealing at 60°C for 15 sec, and extension at 72°C for 10 sec. The plate reading was set after the 72°C step. The amplification cycles were followed by a melt curve set from 70°C to 100°C, with 0.5°C increment every 5 seconds. Primers used were the following: IL-6, F: GAACAACGATGATGCACTTGC and R: TCCAGGTAGCTATGGTACTCC; TNF-*α*, F: AATGGGCTCCCTCTCATCAGTTC and R: TCTGCTTGGTGGTTTGCTACGAC; and GAPDH, F: GGGGCTCTCTGCTCCTCCCTG and R: CGGCCAAATCCGTTCACACCG. Negative control (water instead of DNA) was used to check for nonspecific amplification. Normalized fold expression relative to the control was calculated and plotted using Bio-Rad CFX Manager to compare differential gene expression.

### 2.11. Tissue Fixation, Paraffin Embedding and Haematoxylin and Eosin (H&E) Staining

Kidney tissues were fixed in 10% neutral formalin in a biopsy cassette for 2 days at RT, then dehydrated and embedded in paraffin. Tissue sections from paraffin blocks were mounted on glass slides, deparaffinised, rehydrated, and then stained with haematoxylin and eosin. The slides were then examined under a light microscope.

### 2.12. Dihydroethidium (DHE) Staining

The levels of reactive oxygen species (ROS) were assessed using the DHE staining method (Calbiochem, Darmstadt, Germany) for superoxide anions. Briefly, 10 *μ*M of DHE were applied to dewaxed and hydrated paraffin-embedded kidney sections and incubated in a humidified chamber in the dark for 15 min at 37°C, followed by washing with PBS. Cell Nuclei were stained by Bisbenzimide (Hoechst stain). Tissue images were acquired using a laser scanning confocal microscope (LSM710, Zeiss, Germany). Quantification was done on kidney sections obtained from 5 different animals per condition. A total of 3 different fields of view were quantified per section. The “particle analysis” plugin in the ImageJ software (https://imagej.nih.gov/ij/) was used to obtain DHE fluorescence integrated density (FID), which represents ROS generating cells, and the Hoechst FID, which represents total cells in the tissue section. A ratio was calculated for DHE FID/Hoechst FID for each image. Finally, ratios from all images were averaged and results were analyzed by 2-way ANOVA followed by multiple pairwise analysis using Tukey's post hoc test.

### 2.13. Periodic Acid Schiff (PAS) Staining

This dye is commonly used to stain carbohydrates and polysaccharides, such as glycogen, producing a pink color. Briefly, after dewaxing and hydration steps of the paraffin-embedded kidneys, the tissue was incubated with 0.5% periodic acid for 10 minutes followed by the Schiff reagent for 10–20 min and then washed for 5 minutes, according to manufacturer's protocol (Periodic Acid Schiff Stain Kit, Mucin Stain, Abcam, ab150680). Various steps of dehydration with increased percentage of ethanol (75, 95, and 100%) were done, followed by clearing using xylol, and finally slides were mounted using a mounting medium. Slides were then observed using light microscopy.

### 2.14. Trichrome Staining

Trichrome stain is usually used to highlight collagen fibers in a tissue. Briefly, after dewaxing and hydration steps, the tissue was soaked in Bouin solution for 1 hr at 56°C, washed in running tap water, and then rinsed in distilled water, according to manufacturer's protocol (Connective Tissue Stain, Abcam, ab150686). Tissues were then incubated in hematoxylin for 10 min, washed, and then stained in Biebrich scarlet acid fuchsin for 10 min. After washing, sections were differentiated in phosphomolybdic-phosphotungstic acid solution for 10 min, then transferred to aniline blue solution, stained for 5 minutes, and then mounted and observed using light microscopy.

### 2.15. Statistical Analysis

Results are expressed as the mean ± SEM. Statistical comparisons were performed using 2-way ANOVA followed by multiple pairwise analysis using Tukey's post hoc test in order to determine statistical significance. The *p* value was determined and values for *p* < 0.05, *p* < 0.001, and *p* < 0.0001 (∗, ∗∗, and ∗∗∗, resp.) were considered significant. Microsoft Excel and GraphPad Prism software were used to perform statistical analysis.

## 3. Results

### 3.1. Myocardial Infarction Affects the Ejection Fraction, but Not Infarct Size, in an Acute Mouse Model of Smoke Exposure

**Figure 1:**
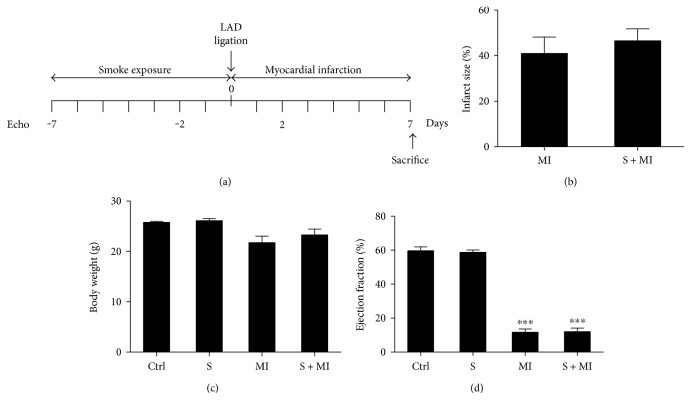
Effect of MI on infarct size (IS), body weight (BW), and ejection fraction percentage (EF%). The experimental model (a) consists of exposing similar age mice to cigarette smoking daily, for 7 days, prior to myocardial infarction (MI) induction via LAD ligation, which also lasted for 7 days. Myocardial infarction had no effect on infarct size (b) or body weights (c). On the other hand, both MI and S+MI groups showed statistically significant decrease in the ejection fraction, in comparison to the S group alone (d). Results are representatives of five different mice in each group (*n* = 5). ∗∗∗ indicate *p* < 0.0001.

This study aimed to mimic the clinical concept of smoking cessation post-MI following hospitalization by investigating the impact of acute smoke exposure on early kidney injury following acute cardiac injury. Indeed, mice were exposed to cigarette smoking daily, for 7 days, prior to myocardial infarction (MI) induction via LAD ligation, which also lasted for 7 days. Animals of similar age (C, S, MI, and S+MI) were used according to an experimental design that mimics acute smoke exposure with or without myocardial infarction (Figure 1(a)). Following the establishment of the model, no difference was found regarding body weights (BW), infarct size (IS) (Figures 1(b) and 1(c)), left ventricular hypertrophy (LVH), or systolic and diastolic blood pressure (data not shown). On the other hand, both MI and S+MI groups showed statistically significant decrease in the ejection fraction percentage (EF%), in comparison to the S group alone (Figure 1(d)).

### 3.2. Effect of Smoking (S) and Myocardial Infarction (MI) on Kidney Histopathology, Using Hematoxylin and Eosin (H&E), Periodic Acid Schiff (PAS), and Trichrome Staining

**Figure 2:**
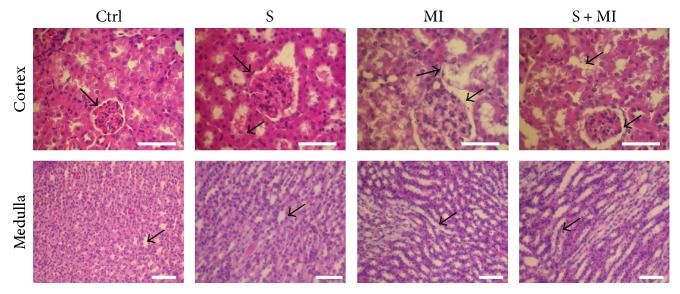
Effect of S and MI on kidney histopathology, using hematoxylin and eosin (H&E) staining. The H&E stain showed a mild and patchy glomerular retraction, with dilation of the proximal convoluted tubules (PCTs), in the S group. MI and S+MI groups showed tubules with patchy dilation of PCTs, focal loss of PCT brush border, thinning of their lining epithelium, and swelling of the cells. Results are representatives of five different mice in each group (*n* = 5). Scale bars are 50 *μ*m.

The H&E stain showed normal cellular glomeruli with preserved tubular structures and absence of any arterial abnormality in the control group (Figure 2 and Table 1). However, a mild and patchy glomerular retraction was found in the smoking (S) group, which is a sign of mild ischemia, with dilation of the proximal convoluted tubules (PCTs). On the other hand, the myocardial infarction (MI) group showed normal glomeruli and vessels along with signs of early acute tubular injury (ATN). Indeed, the tubules presented patchy dilation of PCTs, focal loss of PCT brush border, thinning of their lining epithelium, and swelling of the cells. Furthermore, these PCT lesions present in the MI group were also identified in the combined S+MI group. In addition, there was also patchy glomerular retraction similar to that found in the smoking group. These findings indicate the presence of MI lesions with superimposed glomerular lesions found in the S group (Figure 2 and Table 1).

**Figure 3:**
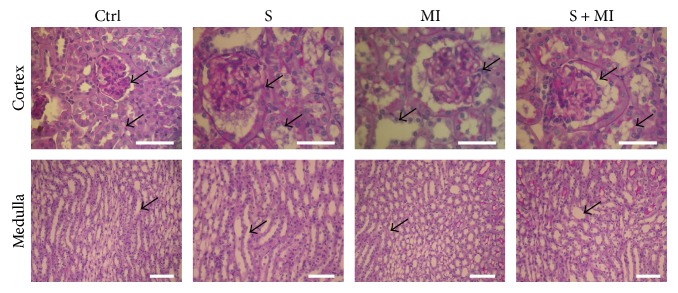
Effect of S and MI on kidney histopathology, using periodic acid Schiff (PAS) staining. The PAS stain revealed dilation of the PCTs while preserving PCT brush borders, in the smoking (S) group. However, MI and S+MI groups showed prominent focal loss of the brush border of PCTs. Results are representatives of five different mice in each group (*n* = 5). Scale bars are 50 *μ*m.

The PAS stain also showed preserved tubular structures and absence of any increased mesangial matrix in the control (C) group (Figure 3 and Table 1). However, the smoking (S) group revealed dilation of the PCTs while preserving PCT brush borders. On the other hand, the glomeruli appeared retracted and vessels were normal. Finally, the focal loss of the brush border of PCTs was highlighted in both the MI and S+MI groups (Figure 3 and Table 1).

**Figure 4:**
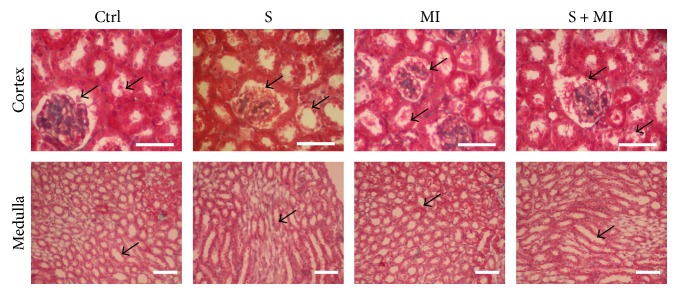
Effect of S and MI on kidney histopathology, using trichrome staining. The trichrome stain confirmed the absence of glomerulosclerosis and interstitial fibrosis in the smoking (S) group. However, the MI group demonstrated swelling of the PCT cells and rare hyaline casts, identified in the tubular lumen. On the other hand, the S+MI group showed PCT dilation with thinning of the epithelial lining and swelling of the cells. Results are representatives of five different mice in each group (*n* = 5). Scale bars are 50 *μ*m.

The trichrome stain showed normal glomerular structure, normal tubules with no interstitial fibrosis or inflammation, and normal vessels in the control group (Figure 4 and Table 1). In addition, the S group confirmed the absence of glomerulosclerosis and interstitial fibrosis. However, trichrome stain demonstrated swelling of the PCT cells in the MI group, as shown by clearing of their cytoplasm, in addition to revealing rare hyaline casts which were identified in the tubular lumen. Finally, trichrome stain showed PCT dilation with thinning of the epithelial lining and swelling of the cells in the S+MI group (Figure 4 and Table 1).

### 3.3. Effect of S and MI on Nephrin and NHE-1 Expression in the Kidney

**Figure 5:**
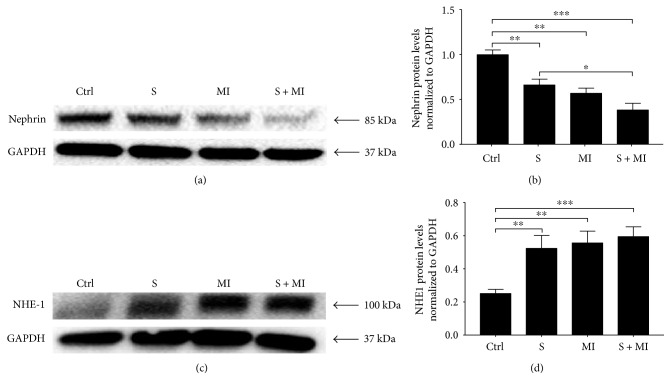
Effect of S and MI on nephrin and NHE-1 protein expression. Western blot analysis showing the effect of S and MI on kidney protein expression of (a) nephrin and (c) NHE-1 markers. (b, d) Histogram analysis showing the relative levels of expression of the above proteins in the 4 groups (C, S, MI, and S+MI). Results showed that S, MI, and S+MI altered protein expression for nephrin and NHE-1. Nephrin expression was significantly decreased in the S+MI group, in comparison to the S group, whereas NHE-1 protein expression increased. Quantification was done by measuring the intensity of each band by densitometry, using ImageJ software. Values represent the average fold change, normalized to GAPDH, and relative to control. Results are representatives of five independent experiments (*n* = 5), for each condition, reported as the mean values ± standard error of the mean (mean ± SEM). ∗, ∗∗, and ∗∗∗ indicate *p* < 0.05, *p* < 0.001, and *p* < 0.0001, respectively.

Nephrin is the first protein identified in slit diaphragms (SD), cytoplasmic extensions of podocytes, and a key molecule to maintain SD structure [39]. Results showed that renal nephrin protein expression was significantly reduced (^∗∗^*p* < 0.001) in S, MI, and S+MI groups, in comparison to controls (Figures 5(a) and 5(b)). This decrease was significantly more prominent in the S+MI group than in the smoking (S) group (^#^*p* < 0.05), indicating that smoking significantly exacerbates MI-induced changes in nephrin expression (Figure 5(b)).

The sodium-hydrogen (Na^+^-H^+^) exchanger-1 (NHE-1), encoded by the SLC9A gene, is a 12-transmembrane protein expressed in renal basolateral proximal tubules and thick ascending epithelia and plays a key role in maintaining homeostatic intracellular volume and pH [40]. Our results showed that S, MI, or S+MI caused a significant (^∗∗^*p* < 0.001) ~2-fold increase in NHE-1 protein levels in the kidney, in comparison to controls (Figures 5(c) and 5(d)).

### 3.4. Effect of S and MI on CTGF, FN, and SMA Expression

**Figure 6:**
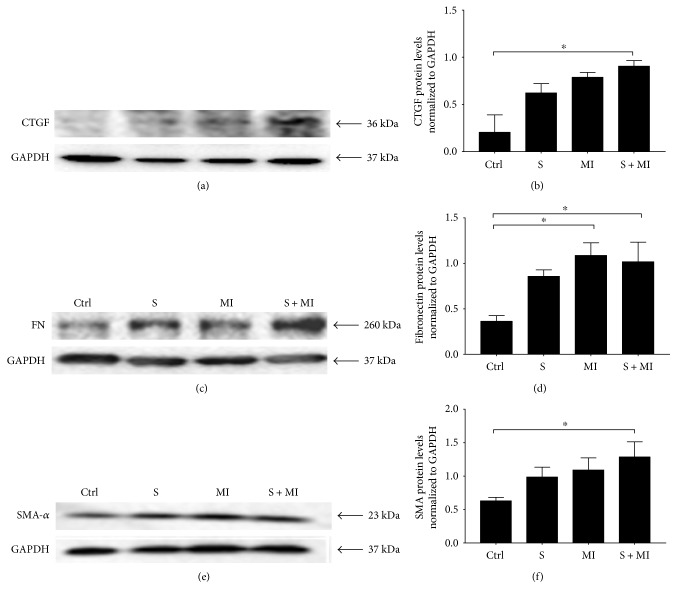
Effect of S and MI on CTGF, FN, and SMA protein expression. Western blot analysis showing the effect of S and MI on the protein expression of (a) CTGF, (c) FN, and (e) SMA markers in the kidney. (b, d, f) Histogram analysis showing the relative levels of expression of the above proteins relative to GAPDH. S+MI group, but not S alone, showed a significant increase in the expression of CTGF, FN, and SMA, in comparison to controls. Quantification was done by measuring the intensity of each band by densitometry, using ImageJ software. Values represent the average fold change, normalized to GAPDH, and relative to control. Results are representatives of five independent experiments (*n* = 5), for each condition, reported as the mean ± SEM. ∗ indicate *p* < 0.05.

Several studies reported high levels of connective tissue growth factor (CTGF), which plays a crucial role in extracellular matrix (ECM) formation leading to kidney fibrosis [41, 42]. In this study, data showed that CTGF renal protein expression increases in both S and MI groups, without reaching statistical significance, in comparison to the control group (Figures 6(a) and 6(b)). However, this increase in CTGF protein expression was statistically significant and further potentiated in the S+MI group, suggesting that S+MI may induce accumulation of ECM proteins, thus contributing to kidney fibrosis.

Similarly, the obtained data showed that S alone had no significant effect on renal protein expression of fibronectin (FN), in comparison to controls, whereas MI and S+MI groups showed a significant increase (^∗^*p* < 0.05) in FN renal protein expression (Figures 6(c) and 6(d)). On the other hand, while S or MI alone did not modify the renal protein levels of smooth muscle actin (SMA), interestingly, S+MI evoked a significant (^∗^*p* < 0.05) increase in its expression, in comparison to controls (Figures 6(e) and 6(f)). These results clearly demonstrate a synergistic effect of S and MI in terms of their effect on the expression of CTGF and SMA in the kidney.

### 3.5. Effect of S and MI on IL-6 and TNF-*α* mRNA Expression

**Figure 7:**
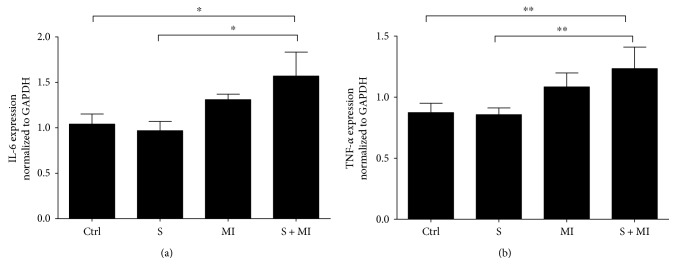
Effect of S and MI on IL-6 and TNF-*α* mRNA expression. Real-Time qPCR showing the effect of S and MI on the mRNA expression levels of IL-6 and TNF-*α*. The S+MI group showed a significant increase in the expression of IL-6 and TNF-*α* mRNA's, in comparison to S group alone or to controls. Data on each target mRNA was normalized to GAPDH. Results are representatives of five different mice in each group (*n* = 5). ∗ and ∗∗ indicate *p* < 0.05 and *p* < 0.001, respectively.

Cigarette smoking is known to cause inflammation and deposition of fibrotic proteins [43, 44]. Therefore, mRNA expression profile of interleukin-6 (IL-6) and tumor necrosis factor-*α* (TNF-*α*) inflammatory markers were assessed in kidneys, using qPCR. Results showed that only the S+MI group had a significant increase in the renal transcriptional expression levels of IL-6 and TNF-*α* (^∗^*p* < 0.05 and ^∗∗^*p* < 0.001, resp.), in comparison to C and S groups (Figures 7(a) and 7(b), resp.).

### 3.6. Effect of S and MI on ROS and NADPH Oxidase-4 (NOX-4) Expression

**Figure 8:**
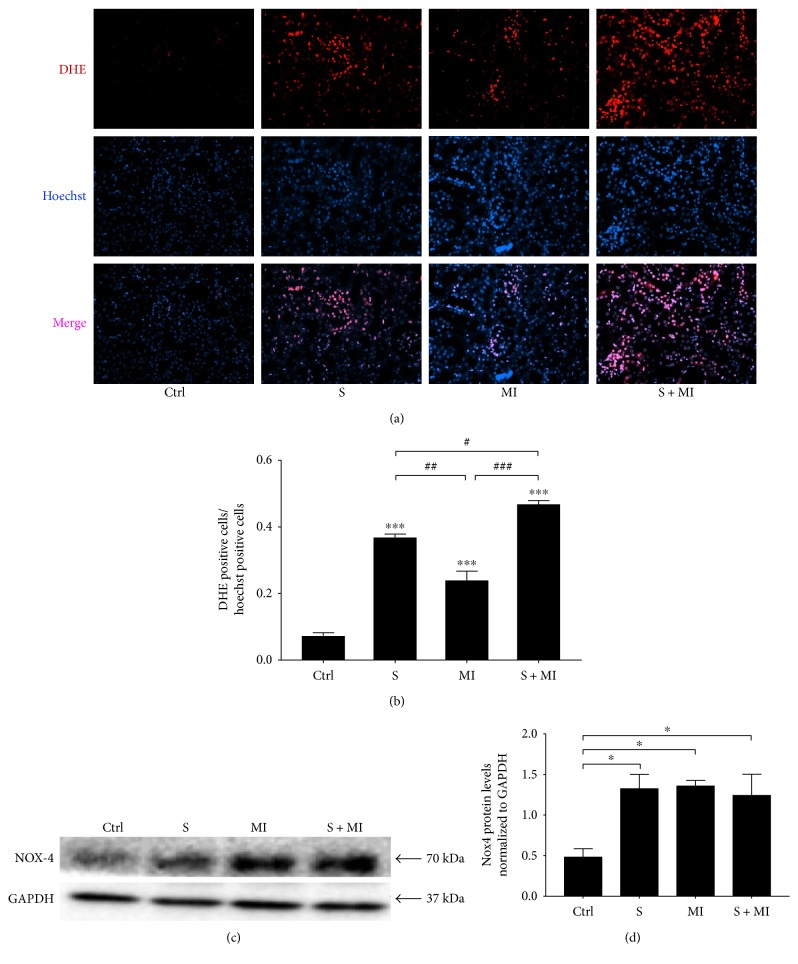
Smoking-induced ROS generation and NOX-4 protein expression in kidneys. (a) DHE staining. Red colour represents DHE, whereas blue refers to Hoechst staining. (b) Quantification of ROS levels. ROS production was drastically increased in the kidneys of S, MI, and S+MI groups, in comparison to controls. The S+MI group showed a more accentuated and prominent ROS production, in comparison to the S or MI groups alone. Results are representatives of five different mice in each group (*n* = 5). All pictures were taken at 40x. (c) The effect of S and MI on NADPH oxidase-4 (NOX-4) protein levels and (d) their quantification in the kidney cortex were evaluated by Western blot. Results showed a significant increase in NOX-4 protein expression levels in S, MI, and S+MI groups, in comparison to controls. Results are representatives of five different mice in each group (*n* = 5). ∗ or #, ∗∗ or ##, and ∗∗∗ or ### indicate *p* < 0.05, *p* < 0.001, and *p* < 0.0001, respectively.

Production of reactive oxygen species (ROS) was then assessed in the different mice groups. Data showed that kidneys in the groups of mice (S, MI, or S+MI) revealed statistically significant increase (^∗∗∗^*p* < 0.0001) of ROS generation, in comparison to controls (Figures 8(a) and 8(b)). In addition, ROS production was more accentuated and prominent in the S+MI group, in comparison to the S or MI groups alone. It is also important to note that S alone caused a significant increase in ROS, in comparison to MI.

It has been reported that kidney injury induces the expression and activation of NOX-4 [45], which plays a major role in ROS generation leading to alterations in signaling pathways related to cellular proliferation, migration, and apoptosis. Since NOX-4 is one of the major sources of ROS in the kidney cortex, its protein expression levels were evaluated by Western blot. Results showed a significant (^∗^*p* < 0.05) increase in NOX-4 protein expression levels in S, MI, and S+MI groups, in comparison to controls (Figure 8(c)).

## 4. Discussion

In this study, we report that myocardial infarction (MI) aggravates the ischemic acute renal damage caused by cigarette smoking (S) leading to renal disintegration and function impairment in kidneys of a mouse model. The above statement is supported by the following: (1) Histological analysis of the cigarette smoking followed by MI (S+MI) group showed alterations in kidney structure such as proximal convoluted tubules (PCTs), swelling of the PCT cells with thinning of the epithelial lining, focal loss of the brush border of PCTs, and patchy glomerular retraction. (2) Cigarette smoking (S), as well as MI, altered nephrin and NHE-1 protein expression, in comparison to controls. (3) Nephrin expression was significantly reduced in the S+MI group, in comparison to the S group, whereas NHE-1 was significantly increased. Thus, cigarette smoking followed by MI (S+MI) induces altered glomerular filtration and kidney functions which leads to kidney fibrosis. (4) Cigarette smoking followed by MI (S+MI) group, but not S alone, showed a significant increase in protein expression levels of CTGF, FN, and SMA, in comparison to controls, suggesting accumulation of ECM. (5) MI and cigarette smoking followed by MI (S+MI) groups showed a significant increase in the mRNA expression levels of inflammatory markers IL-6 and TNF-*α*. (6) ROS production was significantly more accentuated and prominent in the cigarette smoking followed by MI (S+MI) group, in comparison to S or MI groups alone. In fact, a significant increase in NOX-4 protein levels was demonstrated in S, MI, and S+MI groups, in comparison to controls.

In this study, nephrin expression was significantly reduced in the cigarette smoking followed by MI group, implying that MI worsens cigarette smoking induced renal damage. Nephrin is required to maintain slit diaphragm (SD) integrity and PI3K/AKT signaling, which provides a strong survival signal protecting renal cells from stress-induced death [46]. Indeed, the interaction between nephrin and the p85 subunit of PI3K leads to PI3K/AKT activation and regulation of gene expression, migration, and cell viability. Cigarette smoking alters the expression of nephrin protein and leads to glomerular abnormalities and SD structure alteration leading to increase of proteinuria, podocytes death, and podocytes depletion. AKT survival pathway downregulation leads to the development of progressive kidney disease, in addition to the inhibition of collagenase, and the induction of laminin and type 4 collagen, both of which are key components of the glomerular basement membrane [47, 48]. In addition, cigarette smoking has been shown to downregulate nephrin expression in several models of chronic kidney diseases [49]. Therefore, S leads to disruption of structural and functional integrity of the SD proteins that usually play a crucial role in preventing apoptosis and enhancing cell survival [50, 51]. The results from this study confirm previously published reports about downregulation of nephrin expression induced by cigarette smoking in addition to showing that nephrin expression is further downregulated when cigarette smoking is followed by MI.

In renal proximal tubules, Na^+^ binding to an extracellular NHE-1 site triggers a conformational change that translocates Na^+^ across the membrane. This structural alteration simultaneously creates a cytoplasmic binding site for intracellular H+, hence permitting H+ binding and export [40]. NHE-1 plays a crucial role in the maintenance of extracellular fluid volume through proximal tubule isosmotic reabsorption of 50–90% of filtered Na^+^. The apoptotic stress, generated by S in the kidney, activates NHE-1 [52]. We have shown in this study that S, MI, and S+MI increase the expression levels of NHE-1 and also they lead to increased ROS levels. It is conceivable that the increase of NHE-1 expression is mediated by the increased ROS levels. In fact, several studies confirmed that ROS production caused an increase in the levels of NHE-1 [53–55]. This increase in NHE-1 levels is thought to be mediated through the ERK1/2 kinase activation [55]. It will be interesting to investigate whether the increase in NHE-1 in our groups of mice (S, MI, and S+MI) is mediated by ERK1/2 activation. Moreover, apoptosis could be at play in the current model and hence future studies should determine whether apoptosis plays a role in elevating the levels of renal NHE-1 upon exposing mice to cigarette smoking, MI, and cigarette smoking followed by MI. In addition, kidney cells undergo cytosol acidification and catalyse proapoptotic enzymes during apoptosis. NHE-1 defends against renal tubular epithelial cell apoptosis by increasing cytosolic pH as well as expanding cell volume. Moreover, anaerobic metabolism causes intracellular acidosis, which triggers NHE-1 expression and activity. Future work is needed to explore the link between NHE expression and hypertension in this experimental acute mouse model.

High extracellular glucose induces CTGF expression in mesangial cells and increases the production of FN and COL4 leading to ECM production [41, 42]. In this study, there was a gradual increase in the expression of CTGF protein expression in the S, MI and S+MI groups, in comparison to controls. In addition, PAS staining showed increased fibrosis in the S and MI samples, which could be due to increased CTGF expression and ECM formation. Furthermore, a significant increase in CTGF protein levels was shown in S+MI group, in comparison to S or MI alone, which indicates that the combination of S+MI further complicates S-induced renal damage.

Smoking induced the expression of IL-6 and TNF-*α* inflammatory genes in the kidneys of MI and S+MI groups. Also, the expression of the fibrotic marker FN was altered, which has an important role in the initiation and progression of atherosclerosis through the promotion of hypertrophy of vascular smooth muscle cells. Many studies have reported a strong association between smoking and CVD occurrence [56–59] whereas others failed to find any association, possibly due to the study population size [42]. Smoking is a worldwide health burden and is associated with harmful health problems, especially on the cardiovascular (CV) system. The exact mechanism by which smoking promotes CVD is not well understood; however, many studies have reported that S induces inflammation, ROS generation, and reactive nitrogen species (RNS), which lead to various complications on the CV system including atherosclerosis, hypertension, cardiac remodelling, and heart attack. Indeed, ROS act as signaling molecules when they are at low concentrations and the H_2_O_2_ component of ROS participates in signaling pathways related to cellular proliferation, migration, and apoptosis [21, 22]. On the other hand, at high concentrations, ROS and RNS can induce oxidative tissue damage and disrupt normal tissue homeostasis leading to disease [60, 61]. A major source of ROS and RNS generation in the kidney is NADPH oxidases and nitric oxide synthase [62]. In this study, we showed that both MI and S increase the expression of NOX-4 protein, which could explain the increased ROS due to S leading to renal damage.

In summary, our results suggest that MI aggravates the acute renal damage caused by cigarette smoking leading to renal damage and function impairment in kidneys of a mouse model.

## Figures and Tables

**Table 1:** Summary for qualitative assessment of histology sections. The MI group demonstrated swelling of the PCT cells and rare hyaline casts, identified in the tubular lumen. On the other hand, the S+MI group showed PCT dilation with thinning of the epithelial lining and swelling of the cells. MI and S+MI groups showed prominent focal loss of the brush border of PCTs. A total of 10 fields were screened per slide, 5 slides per mouse and 5 mice per group. The overall assessment was denoted as (+) for mild, (++) for moderate, and (+++) for severe.

	Ctrl	S	MI	S+MI
Glomerular retraction	−	+	−	+

*Interstitium*				
Dilation of PCTs	−	++	++	++

*Interstitium*				
(i) Thinning of PCT lining	−	−	++	+++
(ii) Focal loss of brush border				
(iii) Swelling of PCT cells				

*Vessels*				
(i) Subintimal fibrosis	−	−	−	−
(ii) Vasculitis				

## References

[1] Heron M. (2013). Deaths: leading causes for 2010. *National Vital Statistics Report*.

[2] Lim S. S., Vos T., Flaxman A. D. (2012). A comparative risk assessment of burden of disease and injury attributable to 67 risk factors and risk factor clusters in 21 regions, 1990-2010: a systematic analysis for the Global Burden of Disease Study 2010. *Lancet*.

[3] Lloyd-Jones D., Adams R., Carnethon M. (2009). Heart disease and stroke statistics—2009 update: a report from the American Heart Association Statistics Committee and Stroke Statistics Subcommittee. *Circulation*.

[4] World Health Organization (2011). *Global status report on noncommunicable diseases 2010*.

[5] Hayman L. L., Meininger J. C., Daniels S. R. (2007). Primary prevention of cardiovascular disease in nursing practice: focus on children and youth: a scientific statement from the American Heart Association Committee on Atherosclerosis, Hypertension, and Obesity in Youth of the Council on Cardiovascular Disease in the Young, Council on Cardiovascular Nursing, Council on Epidemiology and Prevention, and Council on Nutrition, Physical Activity, and Metabolism. *Circulation*.

[6] Ng M., Freeman M. K., Fleming T. D. (2014). Smoking prevalence and cigarette consumption in 187 countries, 1980-2012. *Journal of the American Medical Association*.

[7] Eriksen M. P., Mackay J. M., Schluger N., Islami F., Drope J. (2015). *The tobacco atlas*.

[8] Rodgman A., Perfetti T. (2013). Carcinogens, Tumorigens, and Mutagens vs. Anticarcinogens, Inhibitors, and Antimutagens, Second Edition. *The Chemical Components of Tobacco and Tobacco Smoke*.

[9] Doll R., Peto R., Boreham J., Sutherland I. (2004). Mortality in relation to smoking: 50 years’ observations on male British doctors. *British Medical Journal*.

[10] Orosz Z., Csiszar A., Labinskyy N. (2007). Cigarette smoke-induced proinflammatory alterations in the endothelial phenotype: role of NAD(P)H oxidase activation. *American Journal of Physiology - Heart and Circulatory Physiology*.

[11] Manley A. F. (1997). Cardiovascular implications of smoking: the surgeon general’s point of view. *Journal of Health Care for the Poor and Underserved*.

[12] US Department of Health and Human Services (2010). *How Tobacco Smoke Causes Disease: The Biology and Behavioral Basis for Smoking-Attributable Disease: A Report of the Surgeon General*.

[13] Shah R., Cole J. (2010). Smoking and stroke: the more you smoke the more you stroke. *Expert Review of Cardiovascular Therapy*.

[14] Pryor W. A., Stone K. (1993). Oxidants in cigarette smoke. Radicals, hydrogen peroxide, peroxynitrate, and peroxynitrite. *Annals of the New York Academy of Sciences*.

[15] Kietadisorn R., Juni R. P., Moens A. L. (2012). Tackling endothelial dysfunction by modulating NOS uncoupling: new insights into its pathogenesis and therapeutic possibilities. *American Journal of Physiology - Endocrinology and Metabolism*.

[16] Cai H., Harrison D. G. (2000). Endothelial dysfunction in cardiovascular diseases: the role of oxidant stress. *Circulation Research*.

[17] Mercado C., Jaimes E. A. (2007). Cigarette smoking as a risk factor for atherosclerosis and renal disease: novel pathogenic insights. *Current Hypertension Reports*.

[18] Droge W. (2002). Free radicals in the physiological control of cell function. *Physiological Reviews*.

[19] Mozsik G., Fiegler M., Juricskay I., Mezey B., Toth K. (1992). Oxygen free radicals, lipid metabolism, and whole blood and plasma viscosity in the prevention and treatment of human cardiovascular diseases. *Forum of Nutrition*.

[20] Brownlee M. (2001). Biochemistry and molecular cell biology of diabetic complications. *Nature*.

[21] Patel R. P., Moellering D., Murphy-Ullrich J., Jo H., Beckman J. S., Darley-Usmar V. M. (2000). Cell signaling by reactive nitrogen and oxygen species in atherosclerosis. *Free Radical Biology & Medicine*.

[22] Cooper D., Stokes K. Y., Tailor A., Granger D. N. (2002). Oxidative stress promotes blood cell-endothelial cell interactions in the microcirculation. *Cardiovascular Toxicology*.

[23] Stokes K. Y., Cooper D., Tailor A., Granger D. N. (2002). Hypercholesterolemia promotes inflammation and microvascular dysfunction: role of nitric oxide and superoxide. *Free Radical Biology & Medicine*.

[24] Harrison D., Griendling K. K., Landmesser U., Hornig B., Drexler H. (2003). Role of oxidative stress in atherosclerosis. *The American Journal of Cardiology*.

[25] Vassalle C., Petrozzi L., Botto N., Andreassi M. G., Zucchelli G. C. (2004). Oxidative stress and its association with coronary artery disease and different atherogenic risk factors. *Journal of Internal Medicine*.

[26] Meltem K., Murat U., Mukaddes E. (2009). Effect of resveratrol on tubular damage and interstitial fibrosis in kidneys of rats exposed to cigarette smoke. *Toxicology and Industrial Health*.

[27] Chang S., Joon S., Soo Y. (2016). Nicotine-induced apoptosis in human renal proximal tubular epithelial cells. *PLoS One*.

[28] Al Hariri M., Zibara K., Farhat W. (2016). Cigarette smoking-induced cardiac hypertrophy, vascular inflammation and injury are attenuated by antioxidant supplementation in an animal model. *Frontiers in Pharmacology*.

[29] van Dokkum R. P. E. (2004). Myocardial infarction enhances progressive renal damage in an experimental model for cardiorenal interaction. *Journal of the American Society of Nephrology*.

[30] Remuzzi G., Perico N., Macia M., Ruggenenti P. (2005). The role of renin-angiotensin-aldosterone system in the progression of chronic kidney disease. *Kidney International*.

[31] Nehme A., Marcelo P., Nasser R., Kobeissy F., Bricca G., Zibara K. (2016). The kinetics of angiotensin-I metabolism in human carotid atheroma: an emerging role for angiotensin (1-7). *Vascular Pharmacology*.

[32] Nehme A., Zibara K., Cerutti C., Bricca G. (2015). 8D.07: gene expression analysis and bioinformatics revealed potential transcription factors associated with renin-angiotensin-aldosterone system in atheroma. *Journal of Hypertension*.

[33] Nehme A., Cerutti C., Dhaouadi N. (2015). Atlas of tissue renin-angiotensin-aldosterone system in human: a transcriptomic meta-analysis. *Scientific Reports*.

[34] Orth S. R. (2000). Smoking–a renal risk factor. *Nephron*.

[35] Rossing P., Hougaard P., Parving H. H. (2002). Risk factors for development of incipient and overt diabetic nephropathy in type 1 diabetic patients. *Diabetes Care*.

[36] Júnior E., Fernando U., Elihimas H. C. D. S. (2014). Smoking as risk factor for chronic kidney disease: systematic review. *Jornal Brasileiro de Nefrologia*.

[37] Husari A., Shihadeh A., Talih S., Hashem Y., El Sabban M., Zaatari G. (2016). Acute exposure to electronic and combustible cigarette aerosols: effects in an animal model and in human alveolar cells. *Nicotine & Tobacco Research*.

[38] Feigenbaum H., Wolfe S. B., Popp R. L., Haine C. L., Dodge H. T. (1969). Correlation of ultrasound with angiocardiography in measuring left ventricular diastolic volume. *American Journal of Cardiology*.

[39] Kestilä M., Lenkkeri U., Männikkö M. (1998). Positionally cloned gene for a novel glomerular protein—nephrin—is mutated in congenital nephrotic syndrome. *Molecular Cell*.

[40] Landau M. (2007). Model structure of the Na^+^/H^+^ exchanger 1 (NHE1). *The Journal of Biological Chemistry*.

[41] Richard J., Elif I., George J. (2014). Markers of and risk factors for the development and progression of diabetic kidney disease. *American Journal of Kidney Diseases*.

[42] Cooper R. (2006). Effect of tobacco smoking on renal dunction. *The Indian Journal of Medical Research*.

[43] Ambrose J. (2004). The pathophysiology of cigarette smoking and cardiovascular disease. *Journal of the American College of Cardiology*.

[44] Kristal B. (1998). The participation of peripheral polymorphonuclear leukocytes in the oxidative stress and inflammation in patients with essential hypertension. *American Journal of Hypertension*.

[45] Gorin Y. (2005). Nox4 NAD(P)H oxidase mediates hypertrophy and fibronectin expression in the diabetic kidney. *The Journal of Biological Chemistry*.

[46] Downward J. (1998). Mechanisms and consequences of activation of protein kinase B/Akt. *Current Opinion in Cell Biology*.

[47] Cantley L. (2002). The phosphoinositide 3-kinase pathway. *Science*.

[48] Talts J. (2001). Akt/PKB regulates laminin and collagen IV isotypes of the basement membrane. *Proceedings of the National Academy of Sciences of the United States of America*.

[49] Lan X., Lederman R., Eng J. M. (2016). Nicotine induces podocyte apoptosis through increasing oxidative stress. *PLoS One*.

[50] Ernst J. (1998). Preparation and characterization of an endogenously fluorescent annexin for detection of apoptotic cells. *Analytical Biochemistry*.

[51] Fries J. (1989). Glomerular hypertrophy and epithelial cell injury modulate progressive glomerulosclerosis in the rat. *Laboratory Investigation*.

[52] Wu K., Khan S., Lakhe-Reddy S. (2003). Renal tubular epithelial cell apoptosis is associated with caspase cleavage of the NHE1 Na^+^/H^+^ exchanger. *American Journal of Physiology - Renal Physiology*.

[53] De Giusti V. C., Caldiz C. I., Ennis I. L., Pérez N. G., Cingolani H. E., Aiello E. A. (2013). Mitochondrial reactive oxygen species (ROS) as signaling molecules of intracellular pathways triggered by the cardiac renin-angiotensin II-aldosterone system (RAAS). *Frontiers in Physiology*.

[54] Sabri A., Byron K. L., Samarel A. M., Bell J., Lucchesi P. A. (1998). Hydrogen peroxide activates mitogen-activated protein kinases and Na^+^-H^+^ exchange in neonatal rat cardiac myocytes. *Circulation Research*.

[55] Rothstein E. C., Byron K. L., Reed R. E., Fliegel L., Lucchesi P. A. (2002). H_2_O_2_-induced Ca^2+^ overload in NRVM involves ERK1/2 MAP kinases: role for an NHE-1-dependent pathway. *American Journal of Physiology-Heart and Circulatory Physiology*.

[56] Rodríguez N. (2006). Mechanisms of disease: oxidative stress and inflammation in the pathogenesis of hypertension. *Nature Clinical Practice Nephrology*.

[57] Powell J. (1998). Vascular damage from smoking: disease mechanisms at the arterial wall. *Vascular Medicine*.

[58] Ryohei Y., MD Y. N., Tatsuya S. (2009). Cigarette smoking and progression of IgA nephropathy. *American Journal of Kidney Diseases*.

[59] Kelly V. (2006). Nodular glomerulosclerosis: renal lesions in chronic smokers mimic chronic thrombotic microangiopathy and hypertensive lesions. *American Journal of Kidney Diseases*.

[60] Bhattacharyya A., Chattopadhyay R., Mitra S., Crowe S. E. (2014). Oxidative stress: an essential factor in the pathogenesis of gastrointestinal mucosal diseases. *Physiological Reviews*.

[61] Safiedeen Z., Rodríguez-Gómez I., Vergori L. (2016). Endoplasmic reticulum cross-talk with mitochondria mediates human microparticle-induced endothelial dysfunction. *Antioxidants & Redox Signaling*.

[62] Sharma K. (2016). Obesity and diabetic kidney disease: role of oxidant stress and redox balance. *Antioxidants & Redox Signaling*.

